# COVID-19 and myositis; true dermatomyositis or prolonged post viral myositis?

**DOI:** 10.1186/s12969-021-00570-w

**Published:** 2021-06-10

**Authors:** Nasim Movahedi, Vahid Ziaee

**Affiliations:** 1 Children’s Medical Center, Pediatrics Center of Excellence, Tehran, Iran; 2 Department of Pediatrics, Tehran University of Medical Science, Tehran, Iran; 3 Pediatric Rheumatology Research Group, Rheumatology Research Center, Tehran University of Medical Science, Tehran, Iran

SARS-CoV-2 infection is best known for its respiratory symptoms, but this virus may cause many complications. One of them is connective tissue like diseases such as Juvenile Dermatomyositis (JDM) [1].

During the COVID-19 pandemic we noticed sudden spur of new cases of JDM hospitalized in our center as a tertiary hospital in Iran. From February 2020 to February 2021, eight new cases of JDM were admitted in Children’s Medical Center, Pediatrics Center of Excellence in Iran. During the same period in 5 years ago (2014 to 2019), just 2 to 4 new cases were admitted (Table 1). Mean of age of patients was higher with a high incidence in female during COVID-19 period.

**Table 1:** Comparison No cases of JDM during COVID-19 pandemic with last 5 years

Year	No of cases	Male/Female	Mean age (year)
**2015**	3	0/3	4.4
**2016**	4	3/1	5.4
**2017**	3	2/1	5.5
**2018**	2	1/1	2.5
**2019**	2	1/1	5.5
**2020**^a^	8	2/6	7.9

^a^COVID-19 pandemic

A surge in the incidence of dermatomyositis has been reported during corona virus pandemic in Mumbai, that was limited to adult population [2]. We proposed three hypotheses to explain it.

The first hypothesis is to accept it as a true dermatomyositis. We know infectious agents can trigger dermatomyositis and JDM appears to have seasonal clustering. Infections with parvovirus B19, coxsackievirus B, enterovirus, influenza, group A streptococcus, and toxoplasma have all been documented in association with JDM. Some viruses play a greater role, perhaps SARS-CoV-2 may be a more potent trigger for this disorder.

Type 1 IFN pathway dysregulation has been implicated in the pathogenesis of JDM. Myxovirus resistance protein A is a type I interferon-inducible protein expressed in response to viral infection, it is also expressed in SARS-CoV-2 infection [3]. Deposition of Myxovirus resistance protein A in muscle fibers and capillaries is an early feature of dermatomyositis, occurring before characteristic perifascicular atrophy.

We assume that true dermatomyositis, triggered by SARS-CoV-2, may occur after this infection and its immunopathogenicity is via type 1 IFN pathway. Tanboon et.al commented that 58-year-old COVID-19 patient reported to have myositis may actually have dermatomyositis [4].

The second hypothesis is to consider it as a prolonged post-viral myositis (PVM) that occurs following SARS-CoV-2 infection. Beydon et.al reported MRI documentation of this type of myositis [5].

PVM typically presents with diffuse or multifocal muscle pain and/or rhabdomyolysis. Symptoms typically localize in the gastrocnemius and soleus muscles; however, other muscles may be involved. Symptoms usually begin about 3–7 days after the onset of fever and respiratory symptoms and then resolve within the first week but can persist up to 1 month [6]. Like other complications of COVID-19, this type of PVM may take longer time for remission.

Finally, it might be a Dermatomyositis-like syndrome, which mimics the symptoms of dermatomyositis but is not a real dermatomyositis. Now, we know some hyperinflammation syndromes as complications of COVID-19 infection. The most common of these syndromes is Multisystem Inflammatory Syndrome in Children, which is present as Kawasaki-like syndrome [7]. Dermatomyositis-like syndrome can be another presentation of hyperinflammation syndromes due to COVID-19. In these cases, if the duration of the disease and its complications are less than those we expect from a dermatomyositis, it can be named Dermatomyositis-like syndrome.

In future with follow up of these patients and more investigation, we can discuss better on this subject.

## Data Availability

All data generated or analysed during this study are included in this published article.

## Abbreviation

JDM: Juvenile Dermatomyositis
